# Promoter Methylation Leads to Decreased *ZFP36* Expression and Deregulated NLRP3 Inflammasome Activation in Psoriatic Fibroblasts

**DOI:** 10.3389/fmed.2020.579383

**Published:** 2021-01-22

**Authors:** Matteo Bertesi, Sebastian Fantini, Claudia Alecci, Roberta Lotti, Andrea Martello, Sandra Parenti, Chiara Carretta, Alessandra Marconi, Alexis Grande, Carlo Pincelli, Tommaso Zanocco-Marani

**Affiliations:** ^1^Laboratory of Applied Biology, Department of Life Sciences, University of Modena and Reggio Emilia, Modena, Italy; ^2^Department of Life Sciences, Centre for Regenerative Medicine “Stefano Ferrari”, University of Modena and Reggio Emilia, Modena, Italy; ^3^Laboratory of Cutaneous Biology, Department of Surgical, Medical, Dental and Morphological Sciences, University of Modena and Reggio Emilia, Modena, Italy; ^4^University College London, Institute of Ophthalmology London, London, United Kingdom

**Keywords:** psoriasis, inflammasome, methylation, ZFP36, NLRP3

## Abstract

The mRNA-destabilizing protein tristetraprolin (TTP), encoded by the *ZFP36* gene, is known to be able to end inflammatory responses by directly targeting and destabilizing mRNAs encoding pro-inflammatory cytokines. We analyzed its role in psoriasis, a disease characterized by chronic inflammation. We observed that TTP is downregulated in fibroblasts deriving from psoriasis patients compared to those deriving from healthy individuals and that psoriatic fibroblasts exhibit abnormal inflammasome activity compared to their physiological counterpart. This phenomenon depends on TTP downregulation. In fact, following restoration, TTP is capable of directly targeting for degradation NLRP3 mRNA, thereby drastically decreasing inflammasome activation. Moreover, we provide evidence that *ZFP36* undergoes methylation in psoriasis, by virtue of the presence of long stretches of CpG dinucleotides both in the promoter and the coding region. Besides confirming that a perturbation of TTP expression might underlie the pathogenesis of psoriasis, we suggest that deregulated inflammasome activity might play a role in the disease alongside deregulated cytokine expression.

## Introduction

Psoriasis is an immune inflammatory skin disease that affects 2–3% of the general population. Although psoriasis primarily affects skin and joints, it often associates with several “systemic” comorbidities, including inflammatory bowel disease, obesity, metabolic syndrome, and cardiovascular disease (1, 2). Psoriasis may impact these comorbid conditions through shared genetic risks, environmental factors, or common inflammatory pathways that are coexpressed in psoriasis and target organs. Overall, a vast range of inflammatory factors are produced in psoriasis lesions, and many of these appear to be released into the systemic circulation as a function of severity and extent of skin lesions.

Scientific literature has widely discussed the origin of psoriasis, examining the role of keratinocytes, immune cells, and even if secondarily, the involvement of fibroblasts. In this regard, early studies proposed that psoriatic fibroblasts could proliferate more than healthy fibroblasts (3); to date, such observation has not been definitively validated, while the attention shifted in turn from fibroblasts to keratinocytes (4). In more recent studies, fibroblasts are thought to rather play a role in maintaining the chronic inflammatory state of psoriatic skin and keratinocytes' proliferation (5, 6). In this context, the observations reported in this work are in accordance with these latest articles.

Tristetraprolin (TTP), encoded by the *ZFP36* gene, is a sequence-specific RNA-binding protein capable of recognizing the optimum binding site UUAUUUAUU located in the 3′ untranslated region (UTR) of its target mRNAs, thereby triggering their degradation (7). TTP induces degradation of several mRNAs encoding key players of the inflammatory response such as TNF, IL-23, and other cytokines (8). Its deletion in keratinocytes determines the appearance of psoriatic-like skin lesions in mice (9). Moreover, *ZFP36*^−/−^ mice, lacking TTP protein, have a severe inflammatory phenotype that includes cachexia, dermatitis, autoimmunity, and inflammatory arthritis, while enhanced stability of TTP mRNA protects mice from imiquimod-induced dermatitis (10, 11). In addition, TTP is involved in a number of comorbidities such as arthritis (12), metabolic syndrome (13), diabetes (14), and atherosclerosis (15).

Inflammasomes are high-molecular-weight protein complexes that are formed in the cytosolic compartment in response to danger- or pathogen-associated molecular patterns. The nucleotide-binding domain, leucine-rich repeat/pyrin domain-containing-3 (NLRP3) inflammasome enables the activation of the inflammatory protease caspase-1, leading to proteolytic cleavage, and release of the pro-inflammatory cytokine interleukin 1β (IL1β) (16). NLRP3 inflammasome has been implicated in the pathogenesis of several autoinflammatory conditions (17). Its role in psoriasis remains to be fully clarified, although several reports suggest its involvement (18, 19). The inflammasome also plays a role in a number of psoriasis-related comorbidities such as metabolic syndrome, atherosclerosis, and cardiovascular disease (20).

In this article, we show that TTP is downregulated in fibroblasts derived from the skin of psoriasis patients compared to those obtained from healthy individuals; we provide evidence that these cells display higher inflammasome activity than their normal counterpart, and we show that TTP restoration determines a decrease in inflammasome activation that depends on the ability to directly target for degradation of NLRP3 mRNA, as reported in macrophages by Haneklaus et al. (21). Eventually, in the absence of mutations or genomic loss events that could explain TTP downregulation during the pathogenesis of psoriasis, we provide evidence supporting the idea that *ZFP36* downregulation in psoriasis might depend on epigenetic mechanisms.

## Materials and Methods

### Cell Cultures

Primary human dermal fibroblasts were isolated from psoriasis patients' skin. Briefly, skin biopsies were surgically removed and immediately stored in a sterile test tube containing medium and antibiotics. All samples were collected with a written informed consent of patients, according to the Declaration of Helsinki after approval of the Modena Medical Ethical Committee. Healthy skin biopsies were obtained from waste materials from operating rooms. Dermal fibroblasts were obtained by explant culture and grown in Dulbecco's modified Eagle's medium (DMEM) containing 5% fetal bovine serum (FBS). Unless otherwise specified, “PSO”-labeled dermal fibroblasts are derived from lesional sites.

The spontaneously transformed keratinocyte line HaCaT was kindly provided by Dr. N. Fusenig (DKFZ, Heidelberg, Germany).

HEK293T was purchased from ATCC.

Dermal fibroblasts, HaCaT, and HEK293T were maintained at 37°C in an incubator with a humidified atmosphere of 5% CO_2_. Cells were cultured in DMEM (Euroclone S.p.A., Pero, MI, Italy) containing 10% heat-inactivated fetal bovine serum (Sigma-Aldrich, St. Louis, MO, USA), 2 mM L-glutamine, and 100 μg/ml of penicillin–streptomycin.

### Cloning Strategies

Full-length *ZFP36* cDNA was cloned EcoRI into pcDNA3.1 expression vector (Invitrogen, Carlsbad, CA, USA) as described in Vignudelli et al. (22) to generate the expression vector pcDNA3.1–ZFP36.

The full length NLRP3 3′UTR region was amplified by PCR from cDNA obtained from RNA of HaCaT cells, using the following specific primers:

NLRP3 3′UTR DP (5′-CAGACGCCAGTGTTCTCCG-3′) and NLRP3 3′UTR RP (5′-CAACCAGCTACAAAAAGCATGG-3′).

The amplified fragment was inserted into the pCR2.1-TOPO T/A cloning shuttle vector (Thermo Fisher Scientific, Waltham, MA, USA) and verified by sequencing. This fragment was then excised with KpnI and XhoI, and cloned into pGL3-Promoter vector (Promega, Milan, Italy), previously modified in order to transfer the multiple cloning region downstream of the luciferase reporter gene (kind gift from Prof. Vincenzo Zappavigna) to generate pGL3–NLRP3-3′UTR(610).

To knock down *ZFP36* expression in human fibroblasts, we used the commercially available pGIPZ lentiviral shRNAmir vector, containing a hairpin sequence targeting *ZFP36* (Open Biosystems) as described in Selmi et al. (23). HEK293T human cells were cotransfected with a lentiviral vector containing the shRNA and lentiviral packaging plasmids (pCMV-dR8.74 plasmid, containing *gag, pol* and *rev* genes; and pMD2.G VSV-G plasmid for the envelope) to produce shRNA-carrying lentiviruses. Culture supernatants were collected 48 h after transfection, and lentivirus particles were concentrated using PEG (System Biosciences). Fibroblasts were transduced by the resulting concentrated viral particles (MOI = 4) overnight and after that immediately supplemented with fresh media. After expansion, cells were sorted (MoFlo cell sorter, Beckman Coulter Inc.) by eGFP-positivity, obtaining two homogeneous populations expressing either the empty vector pGIPZ (EV) or pGIPZ shRNA ZFP36 (shZFP36).

To induce overexpression of TTP, we designed a third-generation lentiviral vector (named pRRL-TTP) where the ΔNGFR cDNA of the bicistronic pRRL.ppt.hPGK.hΔNGFR-IRES-EGFP vector was replaced by the full-length *ZFP36* human cDNA into BamHI sites. Viral supernatants were obtained as described above, and efficiency of transduction was evaluated as a percentage of GFP-positive cells by flow cytometry analysis, without further purification steps.

### Western Blot Analysis and Antibodies

Total cell extracts were prepared as follows: cell pellets were homogenized in ice-cold lysis buffer containing protease and phosphatase inhibitors (10 mM Hepes pH 7.9, 0.1 mM EGTA, 5% glycerol, 0.04% PIC, 0.5 mM PMSF, 1 mM Na3VO4, 50 mM NaF) and left on ice for 30 min. Then the samples were centrifuged at 12,000 × *g* for 30 min at 4°C. Collected lysates were then assayed for protein concentration as described by Bradford (1976). Cell extracts were separated by SDS-PAGE and blotted onto nitrocellulose membranes (0.45 μm pores). Membranes were blocked for 1 h at room temperature in 5% milk and incubated overnight at 4°C with primary antibodies diluted in 5% milk in TBST. Protein detection was performed by ECL (enhanced chemiluminescence, Amersham) reaction, according to the manufacturer's instructions. Antibodies used were actin (MAB1501, clone 4, Millipore Corporation, Billerica, MA, USA), vinculin (AB6039, Millipore Corporation, Billerica, MA, USA), NLRP3 (bs10021R, Bioss, Thermo Fisher Scientific, Waltham, MA, USA), IL-1β (ab34837, Abcam, Cambridge, UK), caspase-1 (#2225S, Cell Signaling Technologies, Danvers, MA, USA), TTP (#71632S Cell Signaling Technologies, Danvers, MA, USA), ASC (AF3805, R&D systems, Minneapolis, MN, USA), and tubulin (MAB1864, clone YL1/2, Millipore Corporation, Burlington, MA, USA). Densitometric analysis of protein expression was determined using ImageJ software.

### DNA Methylation Analysis

Total DNA was extracted with DNeasy Blood & Tissue Kit (Qiagen, Hilden, Germany) and quantified with Nanodrop 2000 spectrophotometer (Thermo Fisher Scientific). Bisulfite conversion was operated with EpiTect Bisulfite Kit (Qiagen) according to the manufacturer's instructions.

PCR amplification of the regions of interest was achieved with EpiMark Hot Start Taq DNA Polymerase (NEB), according to the manufacturer's instruction. Primers for bisulfite-converted DNA amplification were designed with MethPrimer *in silico* tool (24). Primers used for F1 and F2 PCR are: Fw 1_: 3′-AGGTAGGTAGTTTTTAGAGAATTTT-5′ and Rv 2_: 5′-ACACACACACCAAAAAACATACAA-3′ for F1 fragment; Fw 2_: 5′-AATTTGTTTTTGGGATTGTTTTTGTT-3′ and Rv 1_: 5′-ATAAATAACAATCAAATCCATAATATAA-3′ for F2 fragment. To obtain F2 fragment of Psoriasis#2 sample, a longer fragment was amplified by nested PCR through the following primer couple: Fw3_: 5′-GTTGGGATTATAGGTGTGAGTTATTG-3′ and Rv3_: 5′-ACCTCCAAATCACCAAACTAATCTA-3′. Such fragment was then used as template to amplify the F2 fragment using the previously described primers.

PCR products were ligated into the TOPO vector of TOPO TA Cloning kit (Thermo Fisher Scientific) according to the manufacturer's instruction and transformed into DH5α competent cells. Following extraction of the plasmids by alkaline lysis method (“mini-prep”), positive clones were first validated by restriction analysis; then their methylation profile was evaluated by sequencing.

Quality control analysis of sequencer outputs was executed with the aid of BiQ Analyzer bioinformatic tool (25). Sequences were aligned using Blastn BLAST Algorithm (NCBI).

### Chemicals and Treatments

Fibroblasts were treated with 5-aza-2′-deoxycytidine (5-aza; Sigma-Aldrich) in culture medium at a final concentration of 5 μM for 72 h, and dimethyl sulfoxide (DMSO) was used as negative control.

HaCaT cells were treated with actinomycin D (Act.D) in culture medium at a final concentration of 1 μg/ml for the indicated time.

### RNA Extraction and qRT-PCR

Total RNA was extracted with PureLink RNA mini kit (Invitrogen) and quantified with Nanodrop 2000 spectrophotometer (Thermo Fisher Scientific, Waltham, MA, USA). Of total RNA, 100 ng was used to synthesize cDNA with the High Capacity cDNA Retro-transcription Kit (Invitrogen). Quantitative Real-Time PCR (qRT-PCR) was then conducted using an ABI PRISM 7900 detection system (Applied Biosystems, Foster City, CA, USA). All primers and Taqman strategies used for mRNA amplification were designed by Applied Biosystems. Each cDNA sample was run in triplicate using the Taqman Universal PCR Master Mix (Invitrogen), and glyceraldehyde-3-phosphate dehydrogenase (GAPDH) was used as a reference endogenous control. Quantification of RT-PCR signals was performed using the ΔΔC_T_ relative quantification method, which calculates the relative changes in gene expression of the target gene normalized on an endogenous control and compared with a calibrator sample.

Two different isoforms of NLRP3 transcript have been described in literature: a long isoform and a short isoform lacking the most distal portion of the 3′UTR, which includes the ARE motif indicated as a target site for TTP. NLRP3 qRT-PCR experiments reported in this manuscript did not distinguish NLRP3 isoforms since the Taqman probe exploited (Hs00918082_m1, from Thermo Fisher Scientific) is designed on an exon–exon junction. The probe is able to detect all the isoforms annotated in the RefSeq database but do not discriminate isoforms differing in the 3′UTR sequence. For this reason, quantifications of NLRP3 mRNA level here presented might not be representative of the only TTP-sensitive NLRP3 isoform, while transcript level variations reported remain trustworthy.

### Luciferase Assay

HEK293T cells were plated at a density of 50,000 cells/well the day before transfection in 24-wells plates. Two hundred nanograms of pGL3-based reporter constructs, 200 ng of CMV-b-galactosidase plasmid (Clontech Laboratories, Mountain View, CA, USA), and 10 ng of pcDNA3.1 or pCDNA3.1-ZFP36 were transfected in each well. Transfections were performed using Lipofectamine LTX (Thermo Fisher Scientific) as suggested by the manufacturer's guidelines. After 48 h, cells were harvested, and cell lysates were assayed for luciferase and β-galactosidase activity. Luciferase values were normalized on protein concentration and β-galactosidase activity.

### Microarray Data Analysis

Microarray dataset GDS3539 was downloaded from NCBI GEO public database (26). This dataset contained transcriptome data of healthy and psoriatic skin from patients. Pre-processing of expression data was performed through R statistical software and the Affy analysis package from the Bioconductor repository. After pre-processing, boxplots were generated on expression records obtained by selecting microarray probes of interest.

### Statistical Analysis

Data are presented as mean ± SEM, obtained from three different experiments. Prism Software (Graph Pad Software V8.0) was used to perform statistical analysis. A two-tailed unpaired Student's *t*-test was used for statistical comparisons between two groups, as indicated in figure legends. A value of *P* < 0.05 or less was assumed to indicate a statistically significant difference in the compared parameters. Where depicted, each dot in histograms represents a different sample; different styles for the dots represent different cell lines.

## Results

### Expression of Tristetraprolin and Inflammasome Components in Fibroblasts Deriving From Psoriasis Patients

**Figure 1:**
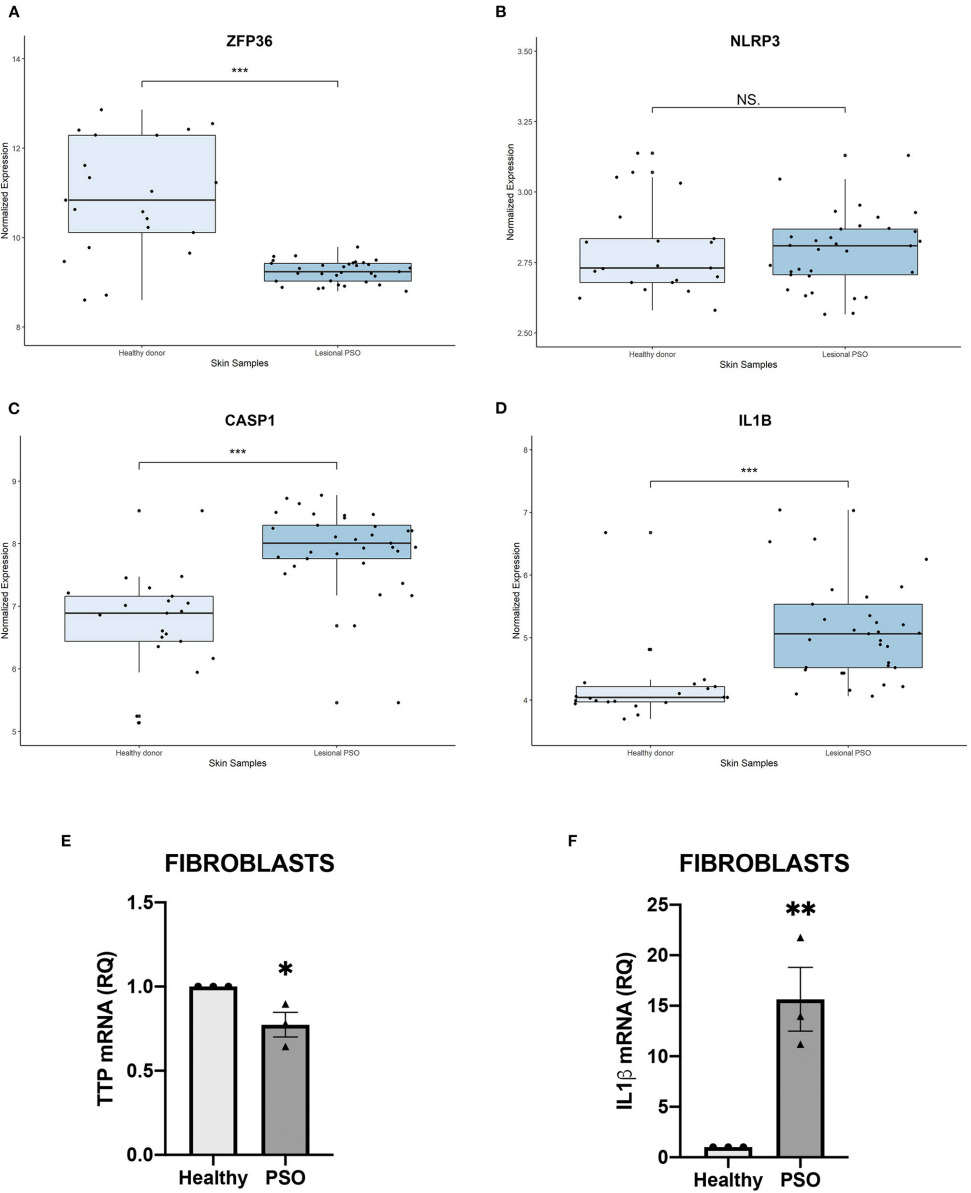
**(A–D)** Relative expression of selected genes, obtained from a publicly available database. Expression of each gene is shown in healthy skin and in lesional psoriatic skin. Boxplots report ±SEM (***p < 0.001). **(A)** *ZFP36* expression in psoriatic skin samples is significantly reduced, compared to healthy skin samples [Healthy donor: *n* = 21, median 10.83, range 8.59–12.85; Lesional psoriatic fibroblasts (PSO): *n* = 33, median 9.23, range 8.79–9.78]. **(B)** *NLRP3* levels show no significant variation among the two groups of healthy and psoriatic skin (Healthy donor: *n* = 21, median 2.73, range 2.58–3.13; Lesional PSO: *n* = 33, median 2.80, range 2.56–3.13). **(C)** *CASP1* expression results increased in lesional psoriatic samples compared to normal skin samples (Healthy donor: *n* = 21, median 6.88, range 5.13–8.52; Lesional PSO: *n* = 33, median 8.00, range 5.45–8.77). **(D)** *IL1B* is significantly more expressed in lesional skin samples from psoriatic patients than skin from healthy donors (Healthy donor: *n* = 21, median 4.04, range 3.69–6.67; Lesional PSO: *n* = 33, median 5.05, range 4.06–7.03). **(E,F)** Relative quantity of mRNA measured by real-time (RT) PCR in healthy and psoriatic fibroblasts samples. **(E)** Differential expression of TTP mRNA in psoriatic and healthy fibroblasts, measured through qRT-PCR. Glyceraldehyde-3-phosphate dehydrogenase (GAPDH) was used as endogenous control. Results are represented as means of three experiments (±SEM) (*p < 0.05). **(F)** IL1β mRNA levels in psoriatic and healthy fibroblasts, measured through qRT-PCR. GAPDH was used as endogenous control. Results are represented as the means of three experiments (±SEM) (**p < 0.01).

In order to verify a possible role of TTP and NLRP3 inflammasome pathway in psoriasis, we initially observed if any variation affecting their mRNAs expression could be detected in psoriatic skin samples. Transcript expression plots obtained from an open-source available dataset (data accessible at NCBI GEO database, ID-REF GDS3539) are presented in Figure 1. *ZFP36* appears substantially downregulated in psoriatic skin samples compared to healthy skin expression level (Figure 1A). *NLRP3* shows no statistically significant variation between the two samples (Figure 1B), although data suggest an increase in its expression in psoriatic skin compared to normal skin. Caspase-1 (*CASP1*) and *IL1*β appear upregulated in psoriatic skin (Figures 1C,D). Relative variations in mRNA levels of TTP and IL1β were then confirmed in our fibroblast samples through real-time PCR, and reported, respectively, in Figures 1E,F.

**Figure 2:**
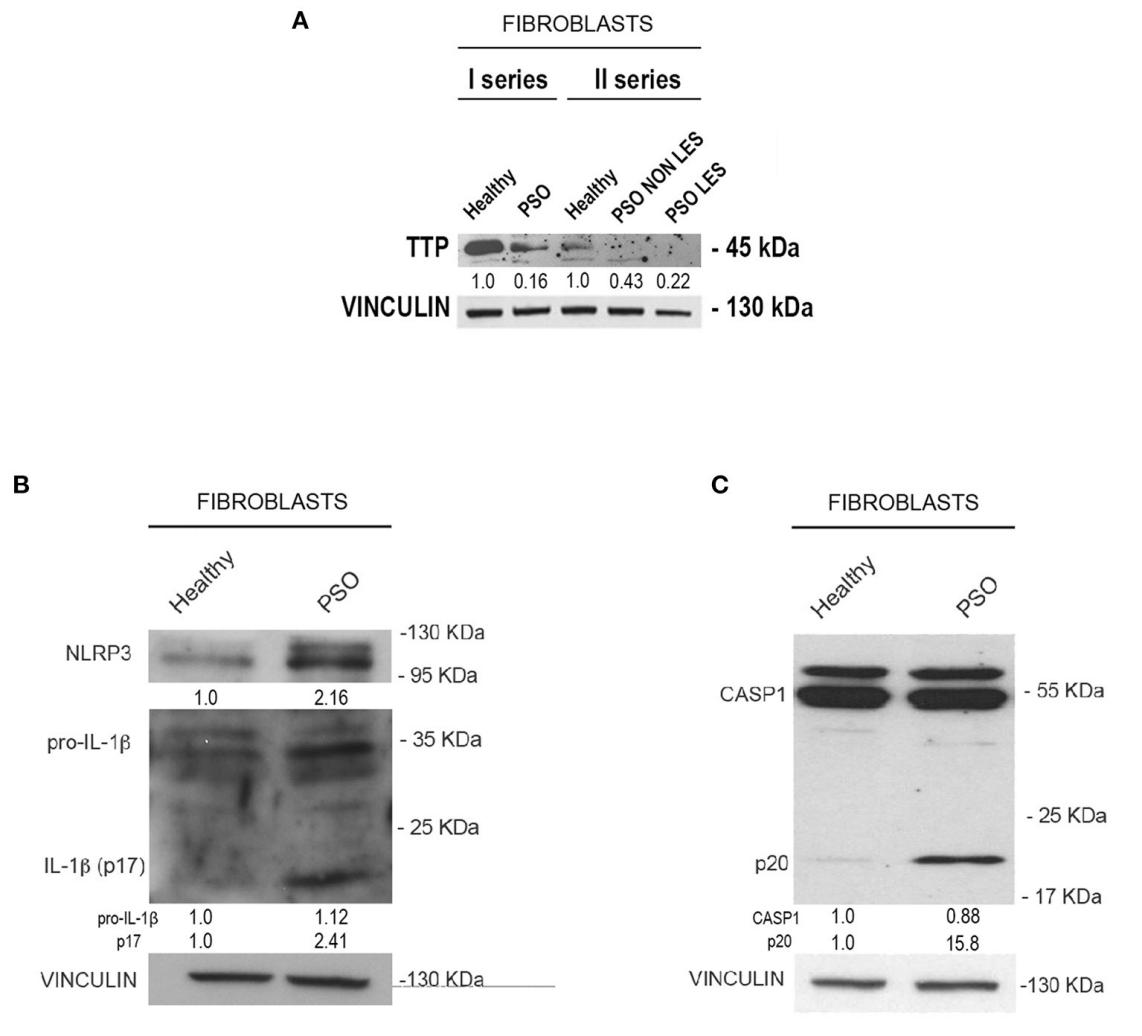
**(A)** Tristetraprolin (TTP) protein levels in healthy and psoriatic fibroblasts. Two different healthy samples and two different psoriatic samples were divided into two series. In “Fibroblasts II series,” psoriatic cell samples from the same donor are distinguished in lesional and non-lesional. Vinculin was used as loading control. **(B)** NLRP3, pro-IL1β, and IL1β (p17) protein levels in healthy and psoriatic fibroblasts. **(C)** Pro-CASP1 and active CASP1 (p20) protein levels in healthy and psoriatic fibroblasts. Vinculin was used in **(B,C)** as a loading control.

Moving from this preliminary overview, we analyzed the expression of TTP and inflammasome main components in fibroblasts deriving from psoriatic donors or healthy individuals. Results of such analysis are shown in Figure 2. In particular, panel A represents a Western blot analysis showing that TTP is significantly downregulated at protein level in psoriatic fibroblasts compared to fibroblasts deriving from healthy donors.

We then analyzed the expression of NLRP3, IL1β and CASP1 in healthy and psoriatic lesional fibroblast samples. Interestingly, as shown in Figures 2B,C, psoriatic fibroblasts show higher levels of NLRP3, higher levels of both pro-IL1β and its active form (p17 subunit), and higher levels of the 20-kDa CASP1 (p20, active form) compared to fibroblasts derived from healthy individuals. Altogether, these data suggest that psoriatic fibroblasts exhibit higher inflammasome activity than their normal counterparts.

### NLRP3 mRNA Is a Direct Target of TTP in Human Fibroblasts

**Figure 3:**
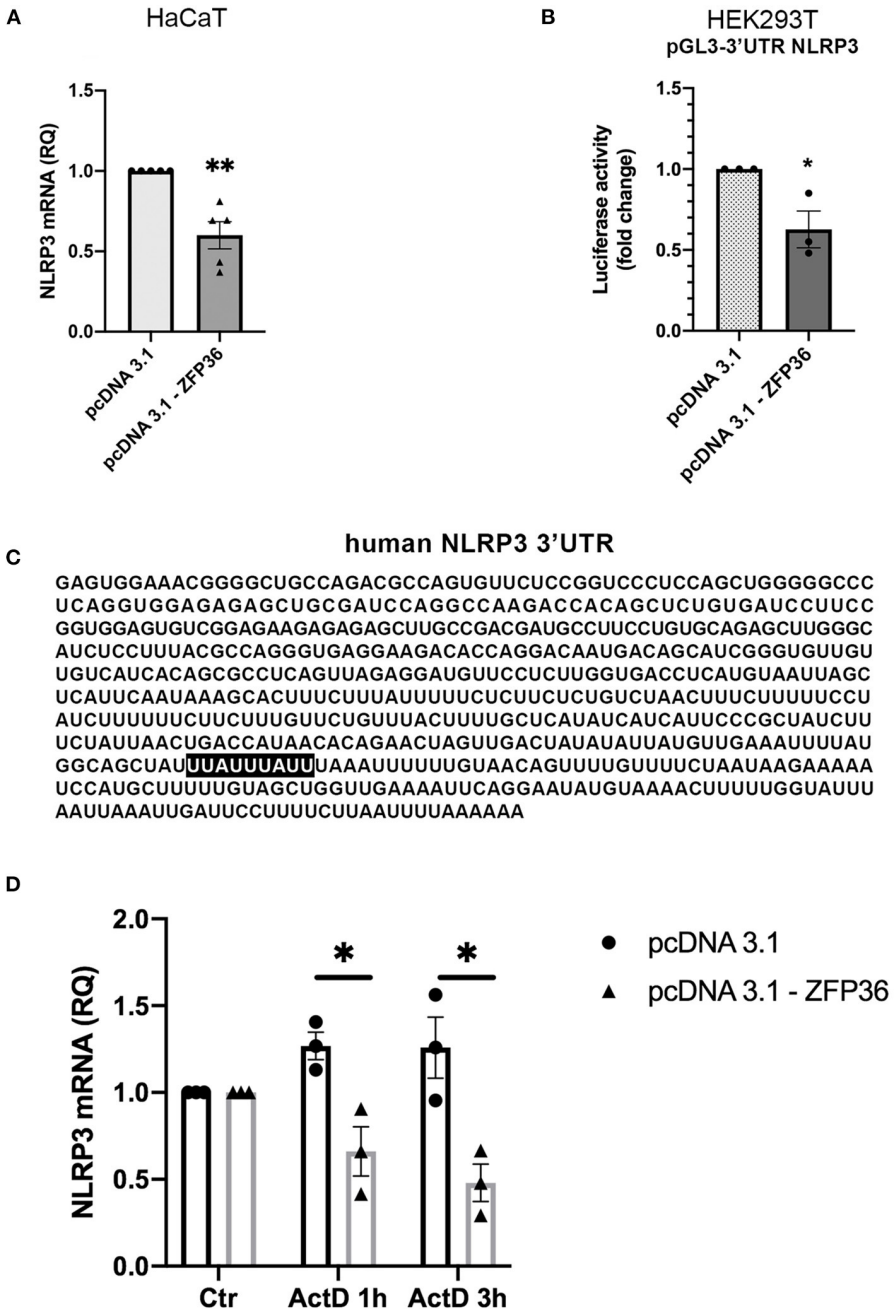
**(A)** qRT-PCR shows a decrease in NLRP3 mRNA in HaCaT cells transfected with a TTP-expressing vector (pcDNA3.1-ZFP36), compared to control transfection (pcDNA3.1). GAPDH was used as endogenous control. Results are represented as the means of three experiments (±SEM) (**p < 0.01). **(B)** Luciferase reporter assay shows a decrease in luciferase activity in HEK293T cell line cotransfected with luciferase reporter vector and the TTP-expressing vector, compared to the control cotransfection with the empty vector. Measured luciferase activity was normalized over β-gal signals. Results are represented as the means of three experiments (±SEM) (*p < 0.05). **(C)** Human NLRP3 3′UTR displaying TTP binding site (highlighted). **(D)** NLRP3 mRNA levels measured by qRT-PCR in HaCaT cells transfected at time 0 with a TTP-overexpressing vector or an empty vector. After transfection (48 h), cells were treated with Act.D to block transcription. NLRP3 mRNA levels were recorded at the moment of ActD treatment (Ctr), after 1 h and after 3 h. Results are represented as the means of three experiments (±SEM). Statistical analysis was performed comparing ZFP36-transfected and empty vector-transfected cells at each timepoint (*p < 0.05). GAPDH was used as endogenous control.

We hypothesized that the inverse correlation between TTP expression and inflammasome activation could depend on the ability of TTP to directly target NLRP3 mRNA for degradation, as it was previously described in macrophages (17). This possibility was supported by the presence of an ARE site specifically bound by TTP on NLRP3 3′UTR, as shown in Figure 3C. To verify this hypothesis, we evaluated the levels of NLRP3 mRNA in HaCaT cell line after transfection with a TTP-expressing vector (named pcDNA3.1-ZFP36) or an empty vector. Cells were collected 48 h after transfection, and NLRP3 messenger levels were analyzed through qRT-PCR, revealing a statistically significant decrease in mRNA quantity upon TTP overexpression (Figure 3A). TTP transfection control can be seen in Supplementary Figure 1A.

To further confirm that NLRP3 is directly targeted by TTP on its 3′UTR, we performed a luciferase reporter assay by cotransfecting in HEK293T cells the vector encoding TTP (pcDNA3.1-ZFP36) or a control vector (pcDNA3.1) with a vector allowing expression of a luciferase cDNA fused to NLRP3 3′UTR sequence [pGL3-NLRP3-3′UTR(610)]. The assay shows that TTP expression causes a significant decrease in luciferase activity compared to cells transfected with the empty vector (Figure 3B).

In addition to the luciferase reporter assay, NLRP3 mRNA half-life was evaluated in HaCaT cells transfected with a TTP-overexpressing vector or an empty vector. To block the synthesis of the nascent mRNA fraction, we treated cells with Actinomycin D (Act.D), a transcription inhibitor. As reported in Figure 3D, Supplementary Figure 1B, HaCaT cells were transfected with either pcDNA3.1-ZFP36 or pcDNA3.1; after 48 h from transfection, each sample was treated with 1 μg/ml Act.D, and NLRP3 mRNA quantity was evaluated in time-course by qPCR. Plotted NLRP3 mRNA relative quantities show a decrease in NLRP3 transcription in HaCaT cells overexpressing TTP compared to the sample carrying the empty vector. To further confirm the role of TTP in the observed NLRP3 mRNA decay, a similar experiment was run, but cells were transfected and treated with Act.D at the same time. In this case no NLRP3 mRNA decrease was detected (Supplementary Figure 1C). These two experiments show that TTP overexpression is able to gradually induce NLRP3 mRNA degradation in HaCaT cells, while the block of TTP expression determined by the treatment with Act.D in proximity of the transfection does not allow NLRP3 degradation. These results suggest that TTP, as previously described in macrophages (17), is capable of triggering degradation of NLRP3 mRNA by binding its 3′UTR sequence.

### *ZFP36* Gene Silencing in Healthy Human Fibroblasts Enhances Inflammasome Activation

**Figure 4:**
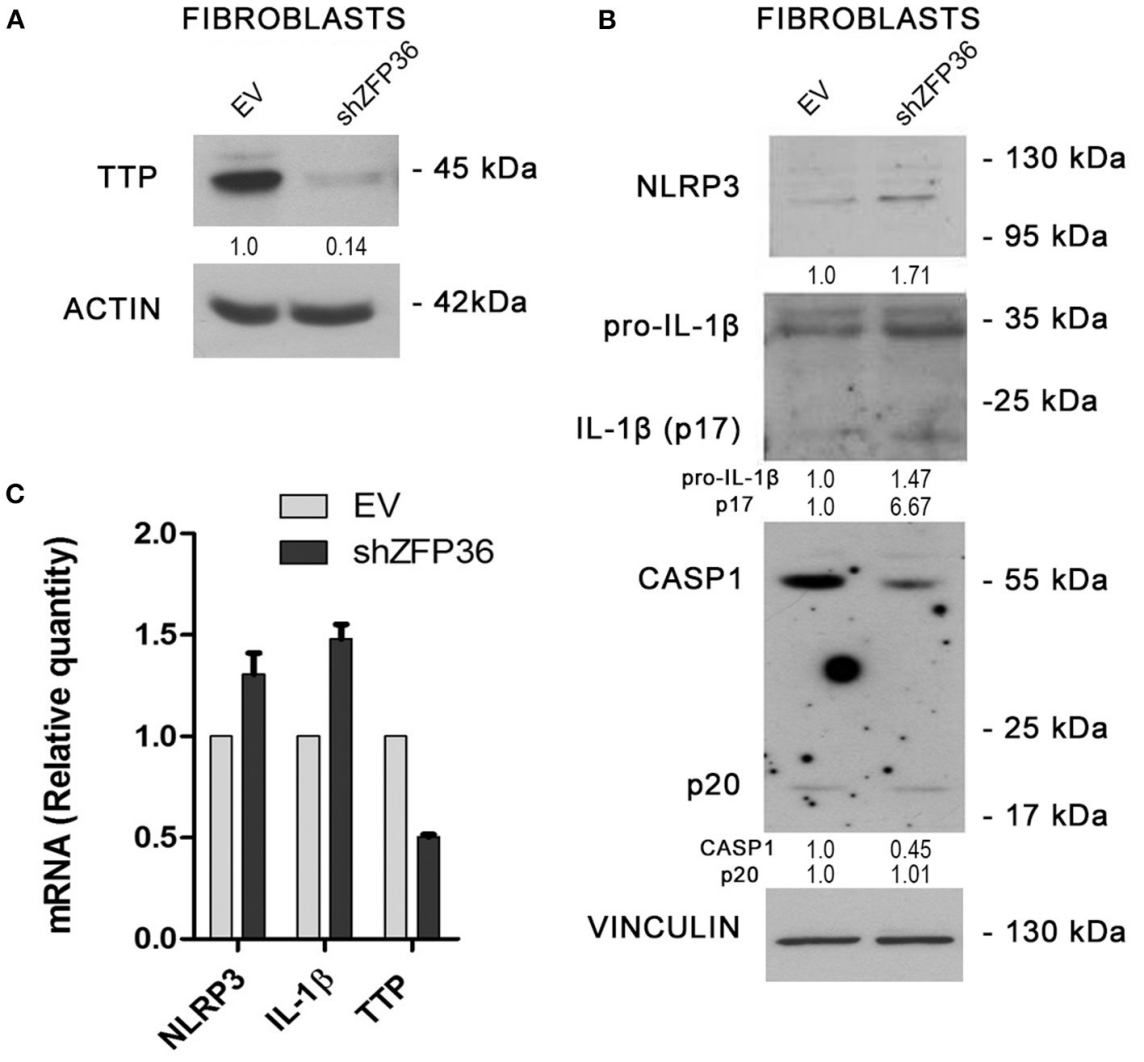
**(A)** TTP silencing achieved by lentiviral infection (sample labeled shZFP36) was verified by Western blot, in comparison to endogenous TTP levels measured after infection with a vector expressing an aspecific scrambled short hairpin (labeled EV). Actin was used as loading control. **(B)** Western blot displaying the expression levels of inflammasome components. Inflammasome interactor levels vary after lentiviral infection with a short hairpin directed against TTP mRNA. Vinculin is included as loading control. **(C)** NLRP3, IL1β, and TTP mRNA levels have been analyzed by qRT-PCR in both cell samples. GAPDH was used as endogenous control. Results are represented as the means of three experiments (±SEM).

Since NLRP3 inflammasome seems more active in psoriatic fibroblasts, which display reduced TTP levels, we evaluated the effects of TTP knock-down on inflammasome activity in fibroblasts from healthy donors. In these cells, *ZFP36* was silenced by means of lentiviral infection by a vector allowing expression of a short hairpin RNA targeting *ZFP36* transcript, as shown in Figure 4A. Successively, we analyzed the expression of the protein complex forming the inflammasome in silenced cells, comparing it to cells infected with the same vector carrying a randomly scrambled short hairpin sequence (EV). Results show that in *ZFP36*-silenced fibroblasts, the expression of NLRP3, pro-IL1β, and IL-1β is increased (Figure 4B), while inactive CASP1 protein level is decreased as a result of activation. The increase in NLRP3 and IL-1β, and the downregulation of TTP were also confirmed by real-time PCR (Figure 4C). Once again, these results confirm in a model of primary cells the inverse correlation between TTP and NLRP3 inflammasome.

### *ZFP36* Ectopic Expression Induces Downregulation of NLRP3 and CASP1

**Figure 5:**
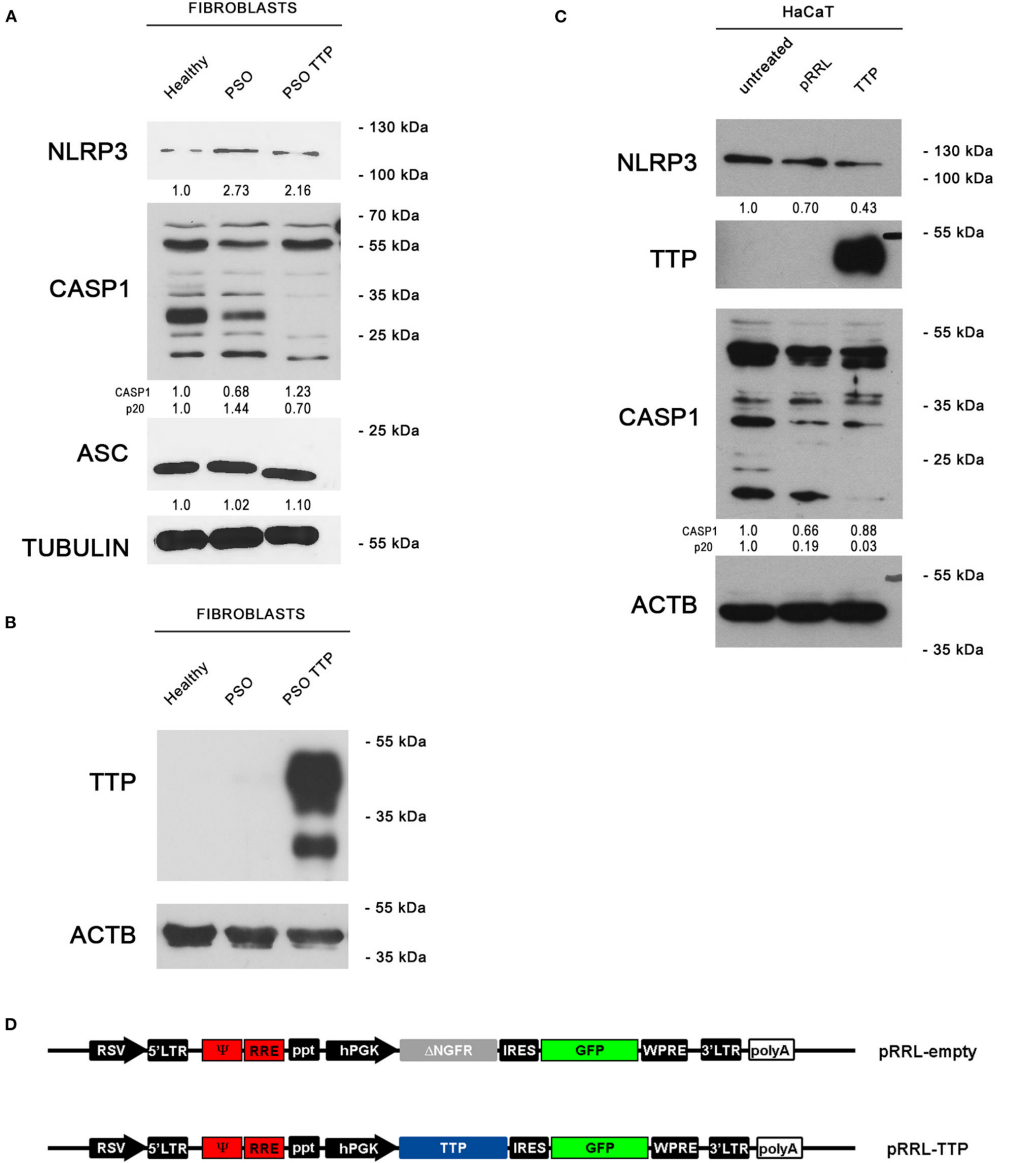
**(A)** NLRP3 inflammasome protein levels are displayed by Western blot in three different fibroblast cell populations: healthy fibroblasts (Healthy), psoriatic fibroblasts (PSO), and psoriatic fibroblasts infected with a pRRL-TTP-overexpressing vector (PSO TTP). Tubulin was used as loading control. **(B)** TTP overexpression is shown in psoriatic fibroblast sample. Actin was used as loading control. **(C)** Inflammasome components variation pattern already seen in primary fibroblasts is maintained in the HaCaT keratinocyte cell line. Actin was used as loading control. **(D)** pRRL-empty vector was used to obtain the overexpression vector pRRL-TTP, by substitution of ΔNGFR sequence with TTP cDNA.

To verify whether the observed variation of TTP expression in psoriatic fibroblasts can potentially affect the activity of NLRP3 inflammasome, we aimed at re-establishing a physiological level of inflammasome components through TTP restoration. To do this, we used a lentiviral vector expressing TTP (pRRL-TTP) using a pRRL-empty vector backbone (Figure 5D). Psoriatic fibroblasts were then infected, and inflammasome component expression in whole-cell extracts of treated psoriatic fibroblasts as well as healthy fibroblasts and untreated psoriatic fibroblasts was analyzed in Western blot. Results are shown in Figure 5A, where NLRP3 protein level in the treated sample is decreased compared to psoriatic control, appearing similar to healthy fibroblast level. Active CASP1 p20 fragment, whose expression is higher in psoriatic cells, is clearly downregulated in the TTP-overexpressing psoriatic sample. Protein level of ASC, adapter protein of NLRP3 inflammasome, is not affected by TTP overexpression. Overexpression of TTP is shown in Figure 5B.

The previous observations were also confirmed in the keratinocyte cell line HaCaT by Western blot (Figure 5C). NLRP3 decrement is present only in the TTP-overexpressing sample compared to untreated cells and empty vector-infected cells. Analogously, p20 CASP1 subunit is detected only in untreated cells or empty vector-infected cells, proving that TTP overexpression negatively regulates inflammasome activation.

### Tristetraprolin Downregulation Observed in Psoriatic Fibroblasts Depends on Methylation Events

**Figure 6:**
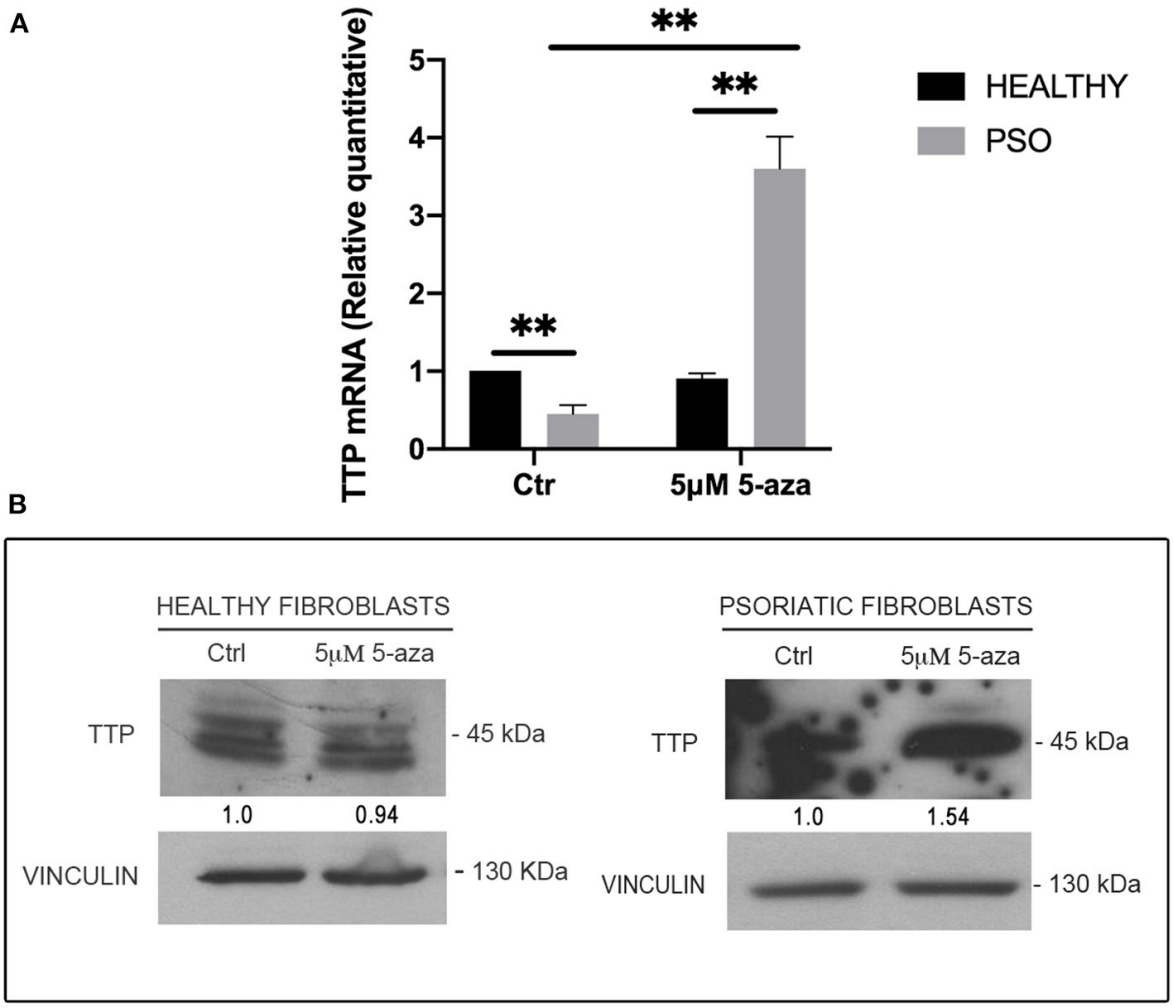
**(A)** TTP mRNA levels in healthy and psoriatic fibroblasts treated with 5-aza, measured by qRT-PCR. GAPDH was used as endogenous control, and results are represented as the means of three experiments (±SEM) (**p < 0.01). Healthy fibroblasts ctr were used as internal calibrator. **(B)** TTP protein levels in healthy and psoriatic fibroblasts after treatment with 5-aza, measured by Western blot. Vinculin was used as loading control.

In order to clarify the causes of TTP downregulation during the pathogenesis of psoriasis, and since no relevant mutation or genomic loss event affecting *ZFP36* has been described in literature, we hypothesized that this occurrence might depend on epigenetic mechanisms. In particular, to verify the involvement of DNA methylation in *ZFP36* regulation, we administered 5-Aza-2′-deoxycytidine (5-aza), a common inhibitor of DNA methyltransferases, to both healthy and psoriatic fibroblasts and then evaluated TTP expression by real-time PCR and Western blot (Figure 6). Figure 6A describes a real-time PCR showing TTP expression in healthy and psoriatic fibroblasts. By comparing healthy and psoriatic 5-aza-untreated fibroblasts (Ctr), one can observe a reduction in TTP expression comparable to that observed in the experiments shown in Figure 2. On the other hand, TTP is strongly increased in psoriasis fibroblasts upon treatment with 5-aza, both compared to healthy 5-aza-treated cells and to psoriasis Ctr population, suggesting a role of methylation underlying ZFP36 silencing in such disease. These results were confirmed in a Western Blot analysis reported in Figure 6B, in which TTP protein quantity increases only in the psoriatic sample treated with methyltransferase inhibitor, while healthy fibroblasts show no sensitivity to 5-aza as regards TTP protein expression.

**Figure 7:**
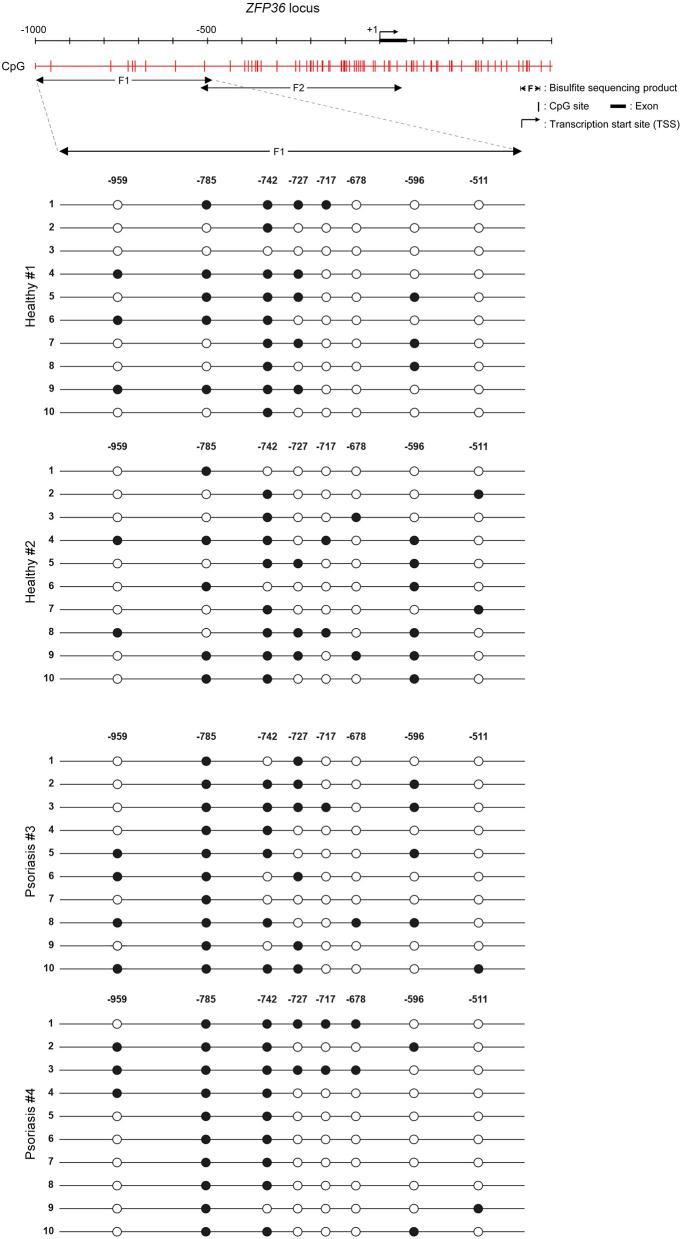
Methylation profile of *ZFP36* promoter in dermal fibroblasts from skin biopsies. CpG sites are represented by red vertical bars along the upstream region of *ZFP36* transcription start site, which was taken as “+1” position of base counting. The “F1” subregion, between −1,000 and −500, was amplified by PCR after bisulfite conversion and then sequenced to obtain the presented methylation profile. Two fibroblast samples from healthy donors and two lesional fibroblast samples from psoriasis donors were analyzed. White circle, unmethylated CpG; black circle, methylated CpG.

The effect of 5-aza on TTP levels in psoriatic fibroblasts supports the hypothesis of a methylation-dependent mechanism for TTP downregulation in psoriasis. For this reason, we evaluated *ZFP36* methylation profile by means of bisulfite treatment. *ZFP36* promoter region was divided into two parts, named F1 and F2. The F1 region, further from transcription start site, presented fewer CpG sites but a higher methylation rate compared to F2. F1 methylation profile (reported in Figure 7) shows a similar occurrence of methylated CpG in healthy control fibroblast samples compared to psoriatic fibroblasts, except for a specific CpG site, called-785, which results more frequently methylated in psoriatic samples compared to the control. This CpG site was methylated with a frequency of 50% in healthy samples compared to a frequency of 100% for the psoriatic samples.

F2 methylation profile, which shows no difference in methylation rate between healthy and psoriatic samples, can be seen in Supplementary Figure 2.

## Discussion

As mentioned in the introduction, TTP seems to be involved in psoriasis and related comorbidities (9–15). In our hypothesis, what these conditions have in common would be an altered TTP expression, leading to an unbalanced production of pro-inflammatory cytokines and determining a persistent inflammation state capable of triggering the abovementioned pathologies.

The inflammasome is a large intracellular complex that, when activated, allows maturation and secretion of IL-18 and IL-1β. Several reports suggest that deregulated activation of NLRP3 inflammasome is involved in arthritis (27), diabetes (28), and chronic diseases such as metabolic syndrome, atherosclerosis, and other age-related diseases (29), and that its altered activity might increase the susceptibility to psoriasis (30). Moreover, several therapeutic agents inhibiting the inflammasome are currently being tested for the treatment of such diseases (31). On these grounds, we hypothesized that TTP, besides regulating the expression of inflammatory factors such as TNFα, could also regulate the inflammasome in fibroblasts, as already observed in macrophages (21). In particular, we focused our attention on NLRP3 inflammasome.

Regarding the cell model in which such possible psoriasis–TTP–inflammasome axis is verified, we decided to use fibroblasts relying on various inflammasome studies performed in this cell type, ranging from cardiac fibroblasts (32) to cancer-associated fibroblasts (33), oral mucosa, lung, and dermal fibroblasts (34–36). Moreover, published data (5, 37, 38) suggest that fibroblasts play an important role in the persistence and in the chronic inflammation state characterizing the disease.

Our hypothesis is also strengthened by the fact that IL-1β has also been described as a direct target of TTP (39). This interaction, together with our hypothesis, outlines a potential “dual regulation” of TTP on inflammasome's activity: on IL-1β mRNA, in order to inhibit its translation, and on NLRP3 mRNA, in order to inhibit the activity of the machinery that leads to cleavage and secretion of IL-1β protein.

Preliminary analyses on publicly available expression data display a significant variation of *ZFP36, CASP1*, and *IL1*β expression level in psoriatic skin samples, compared to healthy skin controls. This data set does not show statistically significant variations in the expression of NLRP3 in psoriatic skin compared to normal skin, although it shows a tendency for higher NLRP3 expression in psoriasis compared to healthy skin.

We focused on fibroblasts, one of the different cell types characterizing skin, and observed that TTP expression at the protein and mRNA level is downregulated in psoriatic fibroblasts compared to healthy ones, as opposed to IL-1β expression, which appears upregulated. Interestingly, TTP is downregulated both in fibroblasts deriving from lesional and non-lesional skin of patients.

Successively, we demonstrated the existence of an inverse correlation between TTP expression and NLRP3 inflammasome activity and confirmed that TTP is capable of directly binding the 3′-UTR region of NLRP3 mRNA, where an AREII sequence specific for TTP binding is present. Altogether, these results demonstrate that TTP seems to be downregulated in psoriasis in humans, accordingly to what was shown by experiments performed on animal models; in addition, it is capable of inhibiting inflammasome activity by directly targeting NLRP3 mRNA, suggesting that deregulation of the inflammasome in psoriasis and its comorbidities might be related to TTP deregulation. To strengthen these hypotheses, we performed a TTP-silencing experiment in healthy fibroblasts through infection of a short-hairpin DNA, which was able to determine a protein expression pattern and mRNA levels of inflammasome components mimicking those already seen in psoriatic fibroblasts.

Last, we needed to understand if overexpression of TTP could counterbalance the defection of TTP observed in psoriasis fibroblasts. Indeed, psoriatic fibroblasts infected with a TTP-overexpressing vector show a rescue of the phenotype of inflammasome protein levels. To further comprehend the reliability of these results, we finally proved that these variations regarding inflammasome protein levels remain reproducible also in a keratinocyte cell line.

Another question that lacks an answer is why TTP is downregulated in psoriasis in the absence of mutations or genomic loss events. In literature, there are reports that TTP expression might be down-regulated in some diseases as a consequence of abnormal activation of upstream pathways (myc, β-cat), while other reports suggest epigenetic causes (40). Indeed, the presence of an extended CpG island in the promoter region of the *ZFP36* gene suggests that TTP expression might undergo methylation events. This possibility seems to be confirmed by an assay performed by administering 5-aza to normal or psoriatic fibroblasts, which determined an increase of TTP mRNA and protein level in psoriatic cells. To confirm the methylation hypothesis, we analyzed the methylation profile of *ZFP36* promoter region through bisulfite sequencing, which revealed an increment in methylation rate of a specific CpG site (named −785C) in psoriatic samples compared to healthy controls. Methylation of −785C could in fact be accountable for the observed decrease in TTP expression by interfering with nearby activators of transcription. Open-source ChIP analysis data available on the Genome Browser revealed the presence of sites for two DNA-binding proteins in the proximity of this CpG site: transcription factor p65 (final effector of NF-κB pathway) with its motif located just downstream of −785C on the minus strand, and POLR2A Polymerase 2 subunit, whose signal begins just upstream of −785C. Notably, the steric hindrance of methyl-binding proteins bound to −785C could easily prevent the downstream factor p65 from deploying Polymerase 2 subunit upstream of −785C. Given the relevance of NF-κB pathway in inflammation signal transduction, it appears clear how p65 could play a predominant role in activating TTP expression in this particular model compared to other transcription factors found in the promoter region. Remarkably, NF-κB autoregulates its effect through a negative feedback loop involving direct induction of TTP expression, thus coupling the trigger and the switching off of the inflammation response. This mechanism has been demonstrated in macrophages (41) and has yet to be confirmed in fibroblasts, but our preliminary data suggest the possibility of an analogous correlation. On the other hand, the negative regulation TTP, which can exert on NF-κB itself and on molecules activating NF-κB signaling, such as TNF and IL-1β, has been thoroughly proven (42–46). In this context, NF-κB impaired activation of TTP expression caused by −785C methylation could be responsible for decoupling inflammation response activation and TTP-mediated inflammation shutdown, outlining the conditions for psoriasis unregulated inflammatory cycle.

Given the role of *ZFP36* in controlling cell cycle and replication (40, 47), such putative TTP downregulation in −785C methylated cells could be accountable for a selective advantage to the expenses of −785C unmethylated cells. This hypothesis is suggested by the increase in −785C methylated fibroblasts in psoriasis samples compared to healthy samples (Figure 7). In psoriasis, inflammation and antiproliferative signals mediated by TTP in physiologically acting fibroblasts could cause a negative selection and the progressive under-representation of such population, promoting the expansion of pathology-related −785C methylated fibroblasts. However, in psoriasis' particular molecular network, where different cell types contribute to symptoms onset, it would be useful to verify whether the observed inverse correlation between inflammasome activity and TTP expression exists also in other contexts, such as keratinocytes and lymphocytes. Moreover, in different pathological cell contexts, where loss of TTP confers a proliferative or survival advantage, it is plausible to hypothesize that TTP silencing can be achieved through different mechanisms.

## Data Availability Statement

The raw data supporting the conclusions of this article will be made available by the authors, without undue reservation.

## Ethics Statement

All samples were collected with written informed consent of patients, according to the Declaration of Helsinki after approval of the Modena Medical Ethical Committee 200703 CVB.

## Author Contributions

TZ-M conceptualized the study. TZ-M, RL, SF, and MB was done by methodology. MB, SF, CA, AMart, and RL performed the investigation. TZ-M and AG sourced resources. TZ-M and MB wrote and prepared the original draft. TZ-M, CP, SP, and CC reviewed and edited the study. AMarc and AG were in charge of project administration. TZ-M acquired funding for the study. All authors contributed to the article and approved the submitted version.

## Conflict of Interest

The authors declare that the research was conducted in the absence of any commercial or financial relationships that could be construed as a potential conflict of interest.
