# Supplementary material for: Promoter Methylation Leads to Decreased ZFP36 Expression and Deregulated NLRP3 Inflammasome Activation in Psoriatic Fibroblasts
Source: Front Med (Lausanne). 2021 Jan 22;7:579383. doi: 10.3389/fmed.2020.579383 (PMC7874095; doi:10.3389/fmed.2020.579383)
Supplement: Supplementary file 1 [file Data_Sheet_1.docx]

**
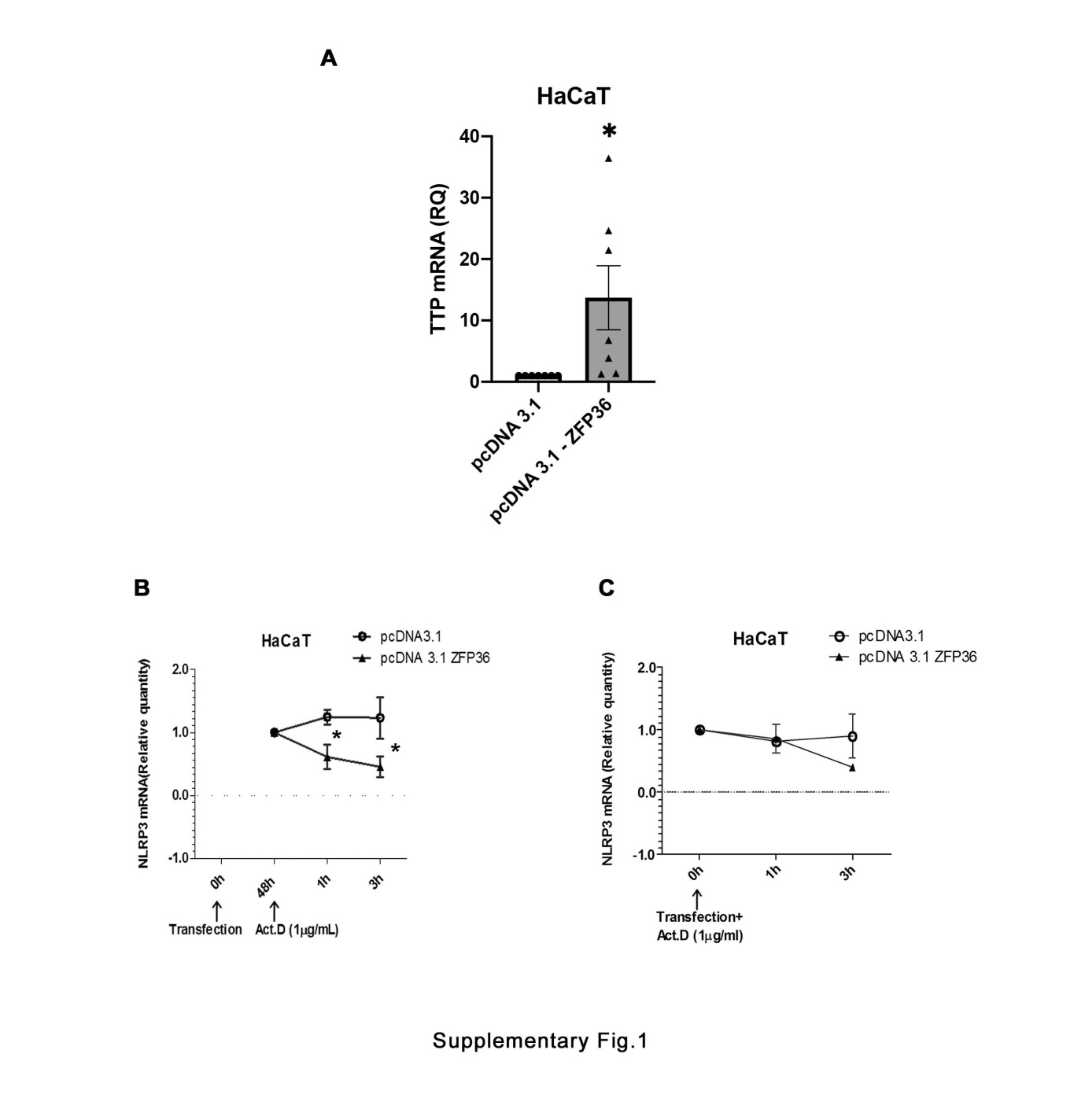
**

**Supplementary Figure 1 -** (**A**) TTP transfection control was performed recording TTP mRNA levels by qRT-PCR in the sample transfected with the empty vector (pcDNA 3.1) and in the sample transfected with the vector overexpressing TTP (pcDNA 3.1 - ZFP36). Results are represented as means of three experiments (+/- SEM)(*p < 0.05). GAPDH was used as endogenous control. (**B**) NLRP3 mRNA levels measured by qRT-PCR in HaCaT cells transfected at time 0 with a TTP-overexpressing vector or an empty vector. 48 hours after transfection, cells were treated with Act.D to block transcription. NLRP3 mRNA levels were recorded in the moment of ActD treatment, after 1 hour, and after 3 hours. Results are represented as means of three experiments (+/- SEM). Statistical analysis was performed between TTP-overexpressing vector and the empty vector transfected cells at each timepoint (*p < 0.05). GAPDH was used as endogenous control. (**C**) NLRP3 mRNA levels measured by qRT-PCR in two different HaCaT cell samples, transfected at time 0 with a TTP-overexpressing vector or an empty vector. At the same time of transfection, cells were treated with Actinomycin D to block cellular transcription as well as vector transcription. NLRP3 mRNA levels were recorded in the moment of ActD treatment, after 1 hour, and after 3 hours. Results are represented as means of three experiments (+/- SEM). GAPDH was used as endogenous control.


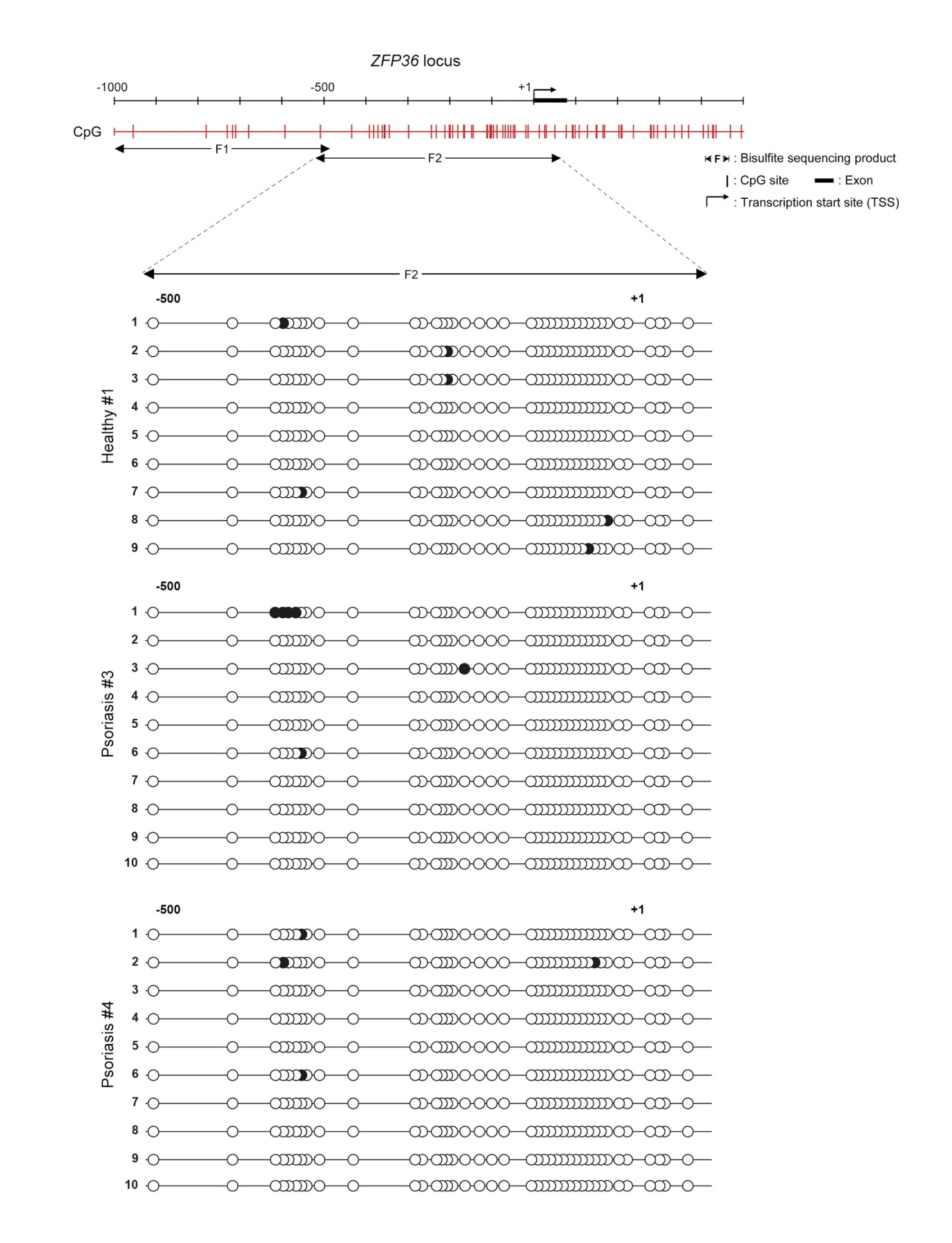


**Supplementary Figure 2 -** Methylation profile of *ZFP36* promoter in dermal fibroblasts from skin biopsies. CpG sites are represented by red vertical bars along the upstream region of *ZFP36* transcription start site, which was taken as "+1" position of base counting. The 'F2' subregion, between -540 and +60, was amplified by PCR after bisulfite conversion and then sequenced to obtain the presented methylation profile. One fibroblast sample from a healthy donor and two lesional fibroblast samples from psoriasis donors were analyzed. White circle, unmethylated CpG; black circle, methylated CpG.
